# MiR-424 and miR-155 deregulated expression in cytogenetically normal acute myeloid leukaemia: correlation with NPM1 and FLT3 mutation status

**DOI:** 10.1186/1756-8722-5-26

**Published:** 2012-06-08

**Authors:** Isabella Faraoni, Serena Laterza, Davide Ardiri, Claudia Ciardi, Francesco Fazi, Francesco Lo-Coco

**Affiliations:** 1 Laboratory of Neuro-Oncohematology, Santa Lucia Foundation, Via del Fosso di Fiorano, 64, Rome, Italy; 2 Department of Neuroscience, University of Rome “Tor Vergata”, Rome, Italy; 3 Department of Biopathology, University of Rome “Tor Vergata”, Rome, Italy; 4 Department of Medico-Surgical Sciences and Biotechnologies, University of Rome “La Sapienza”, Latina, Italy

**Keywords:** Cytogenetically normal AML, MiR-424, MiR-155, NPM1, FLT3-ITD

## Abstract

**Background:**

MicroRNA have a central role in normal haematopoiesis and are deregulated in acute myeloid leukaemia (AML). The purpose of the study was to investigate by qRT-PCR the expression of miRNAs involved in myeloid differentiation (miR-424, miR-155, miR-223, miR-17-5p) in 48 patients with cytogenetically normal AML well characterized for NPM1 and/or FLT3 mutations. Three types of normalization were used for the data validation.

**Findings:**

We found that miR-424 was down-modulated in AMLs with NPM1mutA regardless of FLT3 status. On the contrary, miR-155 showed up-regulation in patients with FLT3 internal tandem duplications (ITD) with or without NPM1 mutations. No significant associations were found by analyzing miR-223 and miR-17-5p in relation to FLT3 and NPM1 status.

**Conclusions:**

This study supports the view that major genetic subsets of CN-AML are associated with distinct miRNA signatures and suggests that miR-424 and miR-155 deregulation is involved in the pathogenesis of CN-AML with NPM1 and FLT3-ITD mutations, respectively.

## Introduction

Acute myeloid leukaemia (AML) is a heterogeneous disease with recurrent cytogenetic alterations detected in approximately 55% of patients, while no karyotypically visible lesions are detectable in the remaining 45% of cases. This latter subset, otherwise referred to as cytogenetic normal AML (CN-AML), is characterized by a variety of subtle mutations affecting several genes. Of these, nucleophosmin (NPM1) alterations account for up to 60% of CN-AMLs and fms-related tyrosine kinase 3 (FLT3) lesions are detected in almost 30% of patients [1,2]. Abnormalities in these genes are not mutually exclusive as they may partially overlap such that four main categories may be identified, i.e. FLT3wt/NPM1wt, FLT3wt/NPM1+, FLT3+/NPM1wt, FLT3+/NPM1 + .

MicroRNAs (miRNAs) are endogenous single-stranded non-coding RNA molecules of 19–24 nucleotides that control gene expression mainly at the post-transcriptional level by binding the 3’untraslated region (UTR) of messenger RNAs to regulate their stability and translation. MiRNAs have emerged as key regulators of normal haematopoiesis and profiling studies have shown altered miRNA expression in leukaemias including AML suggesting their role in leukaemogenesis [3]. However, among the large-scale miRNA profiling studies on AML only few miRNAs were commonly deregulated. Differences in the reported signatures can be attributed to the analysis of distinct cytogenetic and molecular subgroups and to the type of used controls [3,4].

In the present study we focused on CN-AML subsets well characterized for NPM1 and FLT3 status and restricted our analysis to 4 miRNAs known to be involved in normal granulocytic and/or monocytic differentiation (miR-424, miR-155, miR-223, miR-17-5p) [5]. A number of normal controls (CD34+ progenitors, mature granulocytes and monocytes) were also analyzed in order to elucidate whether the expression patterns of the above miRNAs are associated to myeloid differentiation. We found that deregulated expression of miR-424 and miR-155 varies significantly according to NPM1mutA and FLT3-ITD mutational status.

## Design and methods

Fresh primary blast cells were obtained from bone marrow (BM) aspirates of adult patients with newly diagnosed AML admitted at the Department of Biopathology of Tor Vergata University, Rome. All patients provided written informed consent in accordance with the Declaration of Helsinki. BM aspirates with less of 70% of blast infiltration at morphological analysis were discarded. Samples were further enriched for mononuclear cells by Lympholyte Cell Separation Media (Cederlane). FLT3 and NPM1 mutational status was investigated by a multiplex PCR strategy developed in our laboratory and described elsewhere [6].

Samples with FLT3-TKD and nonA type NPM1 mutations were excluded from the study. In order to decrease the level of heterogeneity, only type A mutations for NPM1 and internal tandem duplications (ITD) mutations for FLT3 were included (herein referred to as NPM1+ and FLT3+, respectively). A total of 48 patient samples were selected to identified 4 groups of 12 cases each for the following subsets: FLT3wt/NPM1wt, FLT3wt/NPM1+, FLT3+/NPM1wt, FLT3+/NPM1 + .

Mononuclear cells from BM of healthy control subjects were purified by Lympholyte. CD34+ cells were obtained from cord blood samples and purified by positive selection using MACS immunomagnetic separation system (Miltenyi Biotec). Mature cells were purified from whole peripheral blood of healthy subjects. Granulocytes were recovered and purified by Percoll whereas cells retrieved from the Lympholyte ring were plated and monocytes separated by plastic adherence. The purity of granulocytic (90-95% CD15+, CD16+) and monocytic (78-85% CD14+, CD16-) cell fractions was assessed by flow cytometry.

Total RNA was isolated from fresh cells using Trizol reagent (Invitrogen). All RNA samples were checked for RNA quality by gel electrophoresis. Quantitative real-time PCR (qRT-PCR) of miRNAs was carried out using TaqMan MicroRNA Reverse Transcription Kit and the Taqman MicroRNA primer/probe Assays (Applied Biosystems). Reverse Transcription (RT) reactions were performed using 10 ng of total RNA as detected with NanoDrop ND-1000 spectrophotometer. qRT-PCR reactions, were performed on ABI 7900 HT Sequence Detection System (SDS; Applied Biosystems) and performed in triplicate. The 2^-ΔΔCt^ relative quantification method was used to calculate relative miRNA expression. A small nuclear RNAs (RNU6B) and a small nucleolar (RNU54) commonly employed in miRNA studies were used for internal normalization. The mean value of a normal BM RNA was used as a calibrator in all plates and for all miRNAs.

Group wise comparisons of the distributions of experimental results were performed using unpaired Student t test. All tests were two-tailed, type 3. Results were considered significant for p values equal or below 0.05.

## Results and discussion

**Figure 1:**
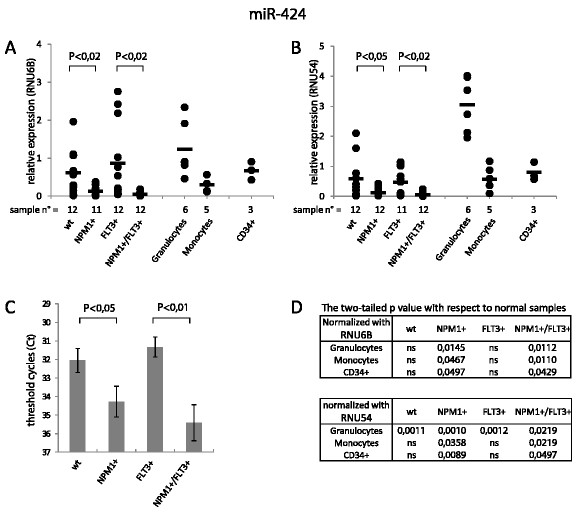
**MiR-424 expression in CN-AML and in normal hematopoietic cells.** MiRNA expression was measured by qRT-PCR using total RNA extracted from fresh samples. CN-AML were subdivided in 4 groups: wt = FLT3wt and NPM1wt; NPM1+=FLT3wt and NPM1mutA; FLT3+=FLT3-ITD and NPM1wt; NPM1+/FLT3+=FLT3-ITD and NPM1mutA. Calibration was obtained with respect to normal BM. (**A**) Relative expression of miR-424 obtained normalizing with respect to RNU6B. (**B**) Relative expression of miR-424 obtained normalizing with respect to RNU54. (**C**) Mean threshold cycles (Ct) of miR-424 in CN-AML was obtained by qRT-PCR. The ordinate scale is represented with the values in reverse order. The error bar indicates the±SE. (**D**) p values of each single group of CN-AML compared to normal samples. ns=not significant.

A significant down-modulation of miR-424 was observed in CN-AML carrying the NPM1 mutation type A (NPM1+). The down-modulation of miR-424 was observed when qRT-PCR data were normalized against both RNU6B (Figure 1A) and RNU54 (Figure 1B). RNU54 has been indicated as the most stable reference gene for miRNAs expression studies in leukaemia samples [7]. However, more recently the use of non-coding RNAs (ncRNA) as internal controls in qRT-PCR reactions has been questioned because they may be deregulated in cancer [8]. In our study, loading of an identical amount (10 ng) of total RNA in the RT reaction (that is given as quantitative by the manufacturer) allowed to evaluate the raw Ct data results for comparisons. The expression of miR-424, related to the total RNA for each sample, was significantly down-modulated in NPM1+ CN-AML (Figure 1C) also in the absence of normalization with a reference gene. As shown in the three panels of Figure 1, miR-424 reduction in NPM1+ cases was not influenced by FLT3-ITD status (FLT3+).

When NPM1+ cases were compared with normal haematopoietic cells we observed a significant down-regulation of miR-424 compared to both differentiated and undifferentiated blood cells, suggesting that such low expression is not related to blast cell immaturity but rather to an aberrant alteration strictly correlated to the NPM1+ expression (Figure 1D).

MiR-424 has been recently classified in a large family cluster together with miR-15/miR-16. Members in this cluster are known to act as tumor suppressors as they can inhibit cell proliferation and promote apoptosis of cancer cells both *in vitro* and *in vivo*[9]. Moreover, miR-424 is known to regulate human myeloid differentiation, at least in part, by blocking translation of the transcription nuclear factor I-A (NFI-A) [10,11]. Our data, showing a reduced miR-424 expression in CN-AML with NPM1+, further support a potential role of this miRNA in leukaemogenesis.

MiR-155 is one of the most studied miRNAs. Sustained expression of miR-155 in haematopoietic stem cells caused myeloproliferative disorders [12]. Moreover, a large number of genes implicated in haematopoietic development and diseases can be directly repressed by miR-155 [13].

**Figure 2:**
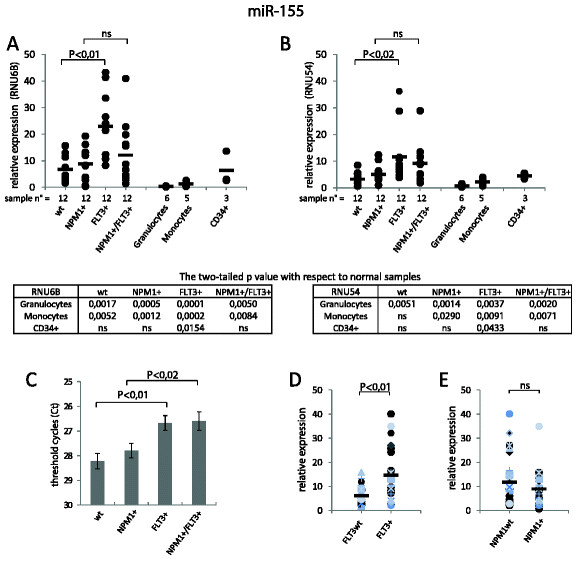
**MiR-155 expression in CN-AML and in human hematopoietic cells.** (**A**) Relative expression of miR-155 was obtained normalizing with respect to RNU6B. (**B**) Relative expression of miR-155 was obtained normalizing with respect to RNU54. (**C**) Mean threshold cycles (Ct) of miR-155 were obtained by qRT-PCR in CN-AML. The ordinate scale is represented with the values in reverse order. The error bar indicates the ± SE. (**D** and **E**) Relative expression of miR-155 was obtained by the geometric mean of RNU6B and RNU54 normalization values. (**D**) CN-AML are subdivided in 2 groups: FLT3wt and FLT3+, each group contains half samples with NPM1wt and half with NPM1+. A significant up-regulation of miR-155 was seen in FLT3+ (ITD-positive) leukemia samples. (**E**) CN-AML are subdivided in 2 groups: NPM1wt and NPM1+, each group contains half samples with FLT3wt and half with FLT3+. No difference in the expression of miR-155 was observed.

As shown in Figure 2A and Figure 2B, we found that miR-155 expression was significantly higher in CN-AML as compared to normal haematopoietic cells. In particular, miR-155 increased expression was associated with FLT3+ mutations in both NPM1wt and NPM1+ samples. However, miR-155 up-regulation was not statistically different when comparing FLT3wt and FLT3+ cases within the NPM1+ groups (Figure 2A and Figure 2B). This finding could rely on normalization over ncRNAs, as previously suggested by the group of Gee et al. [8]. Indeed, evaluation of Ct values showed a statistically significant miR-155 up-regulation in the FLT3+ group regardless of NPM1 status (Figure 2C). It is likely that greater expression differences are not affected by normalization, as in the case of miR-424, in fact, all types of normalization that we showed were equivalent.

To increase the number of cases for comparisons we also analyzed separately FLT3wt *vs* FLT3+ (Figure 2D) and NPM1wt *vs* NPM1+ (Figure 2E) CN-AML. The mean value of miR-155 from RNU6B and RNU54 normalization was used to reduce the bias. A significant up-regulation of miR-155 in FLT3+ samples was detected. The same analysis carried out for miR-155 expression values in CN-AML with respect to NPM1 status disclosed no significant differences.

The up-regulation of miR-155 in AMLs carrying FLT3-ITD is in line with reported findings in CN-AML [14,15]. However, it was also shown by others that miR-155 is slightly up-regulated in CN-AML with NPM1+ [16]. These apparently discrepant data might be better interpreted when considering that, in the study by Ross et al. the NPM1+ subset was considerably enriched in FLT3+ cases (87% of NPM1+ patients). In the present series, comparing the same number of samples for each molecular group we overcame this selection bias, clearly showing that up-regulation of miR-155 is strictly correlated to FLT3+ and not to NPM1+ status.

MiR-223 and miR-17-5p are two additional miRNAs involved in myeloid differentiation [17,18]. By investigating expression levels of these miRNAs in patients with CN-AML, we found no significant association with NPM1 or FLT3 mutation status (data not shown).

In conclusion, we show here for the first time a down-regulated expression of miR-424 in CN-AML and its association with the pathological expression of NPM1+, while we confirm the association of miR-155 up-regulation in CN-AMLs carrying the FLT3-ITD. The classification of miR-155 as an oncomir [19] and the prognostic value of FLT3-ITD in leukemia [1,20] make this association of clinical relevance. These results may foster investigation on the leukaemogenic role of miR-424/miR-155 and on the mechanistic links between key gene mutations and miRNA deregulation in CN-AML.

## Competing interests

The authors declare that they have no competing interests.

## Authors’ contributions

IF carried out miRNA RT-PCR experiments and analyzed the data; SL and CC analyzed NPM1 and FLT3 mutational status; DA was involved in karyotypic studies; IF, FF and FLC designed the study and wrote the manuscript. All authors reviewed and approved the manuscript.
